# Effects of Supplementing Drinking Water of Parental Pigeons with *Enterococcus faecium* and *Bacillus subtilis* on Antibody Levels and Microbiomes in Squabs

**DOI:** 10.3390/ani14020178

**Published:** 2024-01-05

**Authors:** Hui Ma, Yunlei Li, Pengmin Han, Ran Zhang, Jingwei Yuan, Yanyan Sun, Jianhui Li, Jilan Chen

**Affiliations:** 1 State Key Laboratory of Animal Biotech Breeding, Institute of Animal Science, Chinese Academy of Agricultural Sciences, Beijing 100193, China; mahui@caas.cn (H.M.); liyunlei@caas.cn (Y.L.); zhangrancaas@163.com (R.Z.); yuanjingwei@caas.cn (J.Y.); sunyanyan02@caas.cn (Y.S.); 2 Ningxia Xiaoming Agriculture and Animal Husbandry Limited Company, Yinchuan 750000, China; hpm135679@163.com; 3 College of Animal Science, Shanxi Agricultural University, Jinzhong 030800, China

**Keywords:** *Enterococcus faecium*, *Bacillus subtilis*, antibody level, microbiome, probiotics, pigeon milk

## Abstract

**Simple Summary:**

Pigeons are altricial birds; this means that their newly hatched squabs are characterized by closed eyes, little or no plumage, and undeveloped locomotion organs. Pigeon squabs are unable to feed independently and require parental care, especially feeding with pigeon milk in a mouth-to-mouth manner, if they are to survive. The composition of pigeon milk thus plays an important role in the growth of squabs. The results of this study suggest that supplementing the drinking water of parental pigeons with *Enterococcus faecium* and *Bacillus subtilis* increases immunoglobulin levels in pigeon milk. These results provide new insights into the application of probiotics in pigeon production.

**Abstract:**

*Enterococcus faecium* (*E. faecium*) and *Bacillus subtilis* (*B. subtilis*) are widely used as probiotics to improve performance in animal production, but there have been few reports of their impacts on pigeon milk. In this study, twenty-four pairs of parental pigeons were randomly divided into four groups, with six replicates, and each pair feeding three squabs. The control group drank normal water. The *E. faecium* group, *B. subtilis* group, and mixed group drank water supplemented with 3 × 10^6^ CFU/mL *E. faecium*, 2 × 10^7^ CFU/mL *B. subtilis*, and a mixture of these two probiotics, respectively. The experiment lasted 19 days. The results demonstrated that the IgA and IgG levels were significantly higher in the milk of Group D pigeons than in the other groups. At the phylum level, *Fimicutes*, *Actinobacteria*, and *Bacteroidetes* were the three main phyla identified. At the genus level, *Lactobacillus*, *Bifidobacterium*, *Veillonella*, and *Enterococcus* were the four main genera identified. In conclusion, drinking water supplemented with *E. faecium* and *B. subtilis* could improve immunoglobulin levels in pigeon milk, and this could increase the ability of squabs to resist disease. *E. faecium* and *B. subtilis* could be used as probiotics in the pigeon industry.

## 1. Introduction

Different species of birds may be categorized as either altricial or precocial, based on the physiology and anatomy of newborn birds and their maturity of behavior at birth [1]. Pigeon is one of the altricial birds; newly hatched pigeon squabs are characterized by closed eyes, little or no plumage, and undeveloped locomotion organs [1]. Pigeon squabs are unable to feed independently and require parental care, especially feeding with pigeon milk in a mouth-to-mouth manner, if they are to survive [2]. Pigeon milk is a curd-like substance regurgitated from the crops of parental pigeons to their squabs [3]. Pigeon milk, produced by both male and female pigeons, is made up of 60% protein, 32–36% fat, 1–3% carbohydrate, minerals, antibodies, and other nutrients [2,4,5]. Many substances in the pigeon milk of parental pigeons can be transmitted to squabs [6]. Ding et al. (2020) reported that microbiota play important roles in squabs and can be transmitted from parental pigeons to squabs through pigeon milk [2]. Xu et al. (2021) reported that supplementing the diets of parental pigeons with 1% linoleic acid improved intestinal morphology and increased intestinal microbial diversity in their squabs [7]. Researchers have confirmed, then, that supplementation of the diet of parental pigeons may have effects on the growth and health of squabs through its effects on pigeon milk.

Probiotics are beneficial microorganisms that have been shown to inhibit the adherence of pathogenic intestinal bacteria, improve the digestibility of nutrients, and enhance intestinal health [8,9,10]. Researchers have reported that dietary supplementation with probiotic products modulates host-protective immunity against *Clostridium*, *Coliform*, and *Salmonella* infection in both pigs and chickens [11,12,13]. *Enterococcus faecium* (*E. faecium*) is a natural inhabitant of the intestinal tract in poultry; it is known to have positive effects on the growth performance of poultry birds [14,15,16]. Dietary *E. faecium* can increase egg weight and serum FSH levels and reduce levels of *Bacteroidetes* [15]. Dietary supplementation with *E. faecium* has also been found to reduce ammonia emission in laying hens by increasing the digestibility of nutrients [14,17]. Similarly, *Bacillus subtilis* (*B. subtilis*) has been recognized as safe for animal dietary use [18]. It can improve growth performance, balance the intestinal microbiota, enhance immunity, and prevent damage to the intestinal mucosa [19,20].

Antibiotics are traditionally used as therapeutic agents and growth promoters in animal husbandry around the world, but such usage may cause various toxic side effects such as antibiotic resistance in bacteria, drug residues, and damage to intestinal microorganisms, in addition to having adverse impacts upon environmental sustainability [8,9,21]. Because of this, the use of growth-promoting antibiotics has been forbidden in Europe since 2006, and similar bans were imposed in South Korea and China in 2012 and 2020, respectively [22,23]. As a result, studies on such alternative products, such as probiotics, enzymes, and plant extracts, to replace antibiotics have become urgently needed in recent years [24,25]. The effects of probiotics on the growth of pigeon squabs have been reported previously. The addition of a mixture of chitosan oligosaccharide and *Clostridium butyricum* to the diet of pigeon squabs was shown to enhance intestinal health and prevent loss of body weight [26]. In another study, *Bacillus velezensis* isolated from the feces of pigeons enhanced the expression of immune-regulatory genes against pigeon circovirus by means of dietary supplementation with lyophilized *Bacillus velezensis* cells [27]. However, there have been few reports on the effects on squab growth that may result from supplementing the diets of parental pigeons with *E. faecium* and *B. subtilis*. The effectiveness of these probiotics is not yet clear. In this study, therefore, we sought to explore the effects of the supplementation with *E. faecium* and *B. subtilis*, both singly and in combination, in the drinking water of parental pigeons on the growth performance, immunity, intestinal health, and microbiota of their squabs.

## 2. Materials and Methods

### 2.1. Animals and Experimental Design

The viable counts of *E. faecium* and *B. subtilis*, provided by Hebei Cangzhou Huayu Biotechnology Co., Ltd., Cangzhou, China, were 3 × 10^9^ CFU/g and 2 × 10^10^ CFU/g, respectively. White king pigeons were obtained from a commercial farm (Shanxi Red Pigeon Breeding Co., Ltd., Lvliang, China). The experiment was conducted in accordance with the Guide for the Care and Use of Agricultural Animals in Research and Teaching [28]. Slaughter of animals was approved by the Experimental Animal Welfare Ethics Committee of the Institute of Animal Sciences (IAS2021-98).

A total of 24 pairs of parental pigeons were randomly divided into 4 groups, with 6 replicates of each. The “2 + 3” feeding mode was adopted, so that 1 pair of parental pigeons fed 3 squabs, and there were 72 squabs in total. The control group (Group A) drank normal water. The *E. faecium* group (Group B) drank water supplemented with 3 × 10^6^ CFU/mL *E. faecium*. The *B. subtilis* group (Group C) drank water supplemented with 2 × 10^7^ CFU/mL *B. subtilis*. The mixed group (Group D) drank water supplemented with 3 × 10^6^ CFU/mL *E. faecium* and 2 × 10^7^ CFU/mL *B. subtilis*. Supplementation began 7 days prior to the squabs hatching out and continued until the squabs were 12 days old. The experiment therefore lasted 19 days.

### 2.2. Sample Collection and Detection

The body weight (BW) of each squab was measured prior to feeding on Days 1, 3, and 12, and the average daily gain (ADG) was calculated. Samples of pigeon milk were collected from the crops of squabs on Day 3 and placed into sterile centrifuge tubes. Squabs were slaughtered via cervical dislocation after being sedated on Day 12. All milk samples were immediately frozen in liquid nitrogen and then stored at −80 °C for analysis. They were then thawed, and 2 g amounts were used for determination purposes. These were then diluted with 0.9% physiological saline in a 1:9 volume ratio, ground to homogenates, and centrifuged at 10,000 rpm for 20 min. The supernates were collected to determine the levels of immunoglobulin and digestive enzymes. Concentrations of IgA and IgG in pigeon milk were determined using ELISA kits (Beijing Laibo Tairui Technology Development Co., Ltd., Beijing, China) according to the manufacturer’s protocol at a wavelength of 450 nm. Lipase, trypsin, and amylase were measured using commercial assay kits (Beijing Laibo Tairui Technology Development Co., Ltd.) according to the manufacturer’s protocol.

### 2.3. Microbial DNA Extraction and 16S rDNA Sequencing

The pigeon milk was collected and used for bacterial 16S rDNA sequencing. Five samples of pigeon milk from each group were randomly selected for 16S rDNA sequencing. Total bacterial genomic DNA samples were extracted using an OMEGA Soil DNA Kit (Omega Bio-Tek, Norcross, GA, USA) following the manufacturer’s instructions. A NanoDrop NC2000 spectrophotometer (Thermo Fisher Scientific, Waltham, MA, USA) and agarose gel electrophoresis were used to measure the quantity and quality of the DNA. The V3–V4 region of the bacterial 16S rDNA gene was amplified using the forward primer 338F (5′-ACTCCTACGGGAGGCAGCA-3′) and the reverse primer 806R (5′-GGACTACHVGGGTWTCTAAT-3′) [29]. The PCR components contained 4 μL of buffer (5×), 0.4 μL of FastPfu DNA polymerase, 2 μL (2.5 mM) of dNTPs, 0.8 μL (10 μM) of each forward and reverse primer, 1 μL of DNA template, and 11 μL of ddH_2_O. PCR was carried out as follows: initial denaturation at 95 °C for 3 min, followed by 27 cycles consisting of denaturation at 95 °C for 30 s, annealing at 55 °C for 30 s, and extension at 72 °C for 30 s, with a final extension of 10 min at 72 °C. The PCR products were visualized using electrophoresis on 2% agarose gel, then purified with an AxyPrep DNA Gel Extraction Kit (Axygen Biosciences, Union City, CA, USA). Finally, the sequencing of 16S rDNA was performed using an Illlumina NovaSeq platform with a NovaSeq 6000 SP Reagent Kit (500 cycles) at Shanghai Personal Biotechnology Co., Ltd. (Shanghai, China), and purified amplicons were paired.

Sequence data of samples were analyzed using QIIME2 and R packages (version 3.2.0). A number of operational taxonomic units (OTUs) were determined based on 97% similarity using UPARSE (version 7.1). The alpha diversities of the microbiota of pigeon milk and rectal content, including the Chao1 richness estimator, observed species, the Shannon diversity index, Simpson’s index, Faith’s PD, Pielou’s evenness index, and Good’s coverage index, were calculated using the ASV table in QIIME2 to investigate the richness and diversity of the community. Beta diversity analysis was performed to investigate the structural variation in microbial communities across principal coordinate analysis (PCoA), based on the Bray–Curtis matrix. A Venn diagram was generated using the R package [30].

### 2.4. Statistical Analysis

Significant differences among the 4 treatment groups were compared using Tukey’s multiple-comparison test following one-way ANOVA. Differences were considered significant when the *p*-value was less than 0.05. All the statistical analyses were carried out using SPSS 26.0 (IBM, Armonk, New York, NY, USA).

## 3. Results

### 3.1. Growth Performance

The effects of supplementing the drinking water of parental pigeons with *E. faecium* and *B. subtilis* on the growth performance of squabs, including BW and ADG, are shown in Table 1. The results showed that these microbes boosted BW on Day 12 and ADG from Day 1 to Day 12, but the differences were not significant (*p* > 0.05). Meanwhile, parental pigeons drank 430 mL of water in one day in summer.

### 3.2. Determination of Enzymatic Activity and Immunoglobulin Levels in Pigeon Milk

The effects of *E. faecium* and *B. subtilis* on digestive enzyme activity in pigeon milk are shown in Table 2. The activities of lipase, trypsin, and amylase were recorded so that the utilization and absorption rate of pigeon milk could be determined. The results in Table 2 show that supplementation with *B. subtilis* increased the activity of lipase compared with supplementation with *E. faecium*. A mixed supplementation with these two probiotics improved the activity of lipase, as can be seen in the results for Group D, but this did not reach the significance level, compared with that of Group A (*p* > 0.05). With respect to trypsin, mixed supplementation with *E. faecium* and *B. subtilis* significantly improved activity in Group D compared with that in Group B (*p* < 0.05), but the difference was not significant compared with that of Group A (*p* > 0.05). In terms of amylase activity, the value obtained for Group D was significantly higher than that for Group B (*p* < 0.05), but not significantly higher than that for Group A (*p* > 0.05). In conclusion, we found that supplementation with *B. subtilis* improved digestive activity more effectively than supplementation with *E. faecium*, and that a combination of these two probiotics was more effective than supplementation with one or the other alone or no supplementation at all (Table 2).

In the present study, the immunity of pigeon milk was indicated by concentrations of IgA and IgG. Our results showed that concentrations of IgA in Groups B, C, and D were all higher than in Group A. The concentration was significantly 2.28 times higher in Group D than in Group A (*p* < 0.05, Table 3). For IgG, the effect of supplementation with *B. subtilis* was also greater than supplementation with *E. faecium*, and the value for Group D was 2.12 times higher than that for Group A (*p* < 0.05, Table 3). These results suggested that supplementing the drinking water of parental pigeons with *E. faecium* and *B. subtilis* could increase the immunity of pigeon milk, and further suggested that squabs fed with such milk would have better ability to resist disease.

### 3.3. Microbiota Analysis of Pigeon Milk

**Figure 1:**
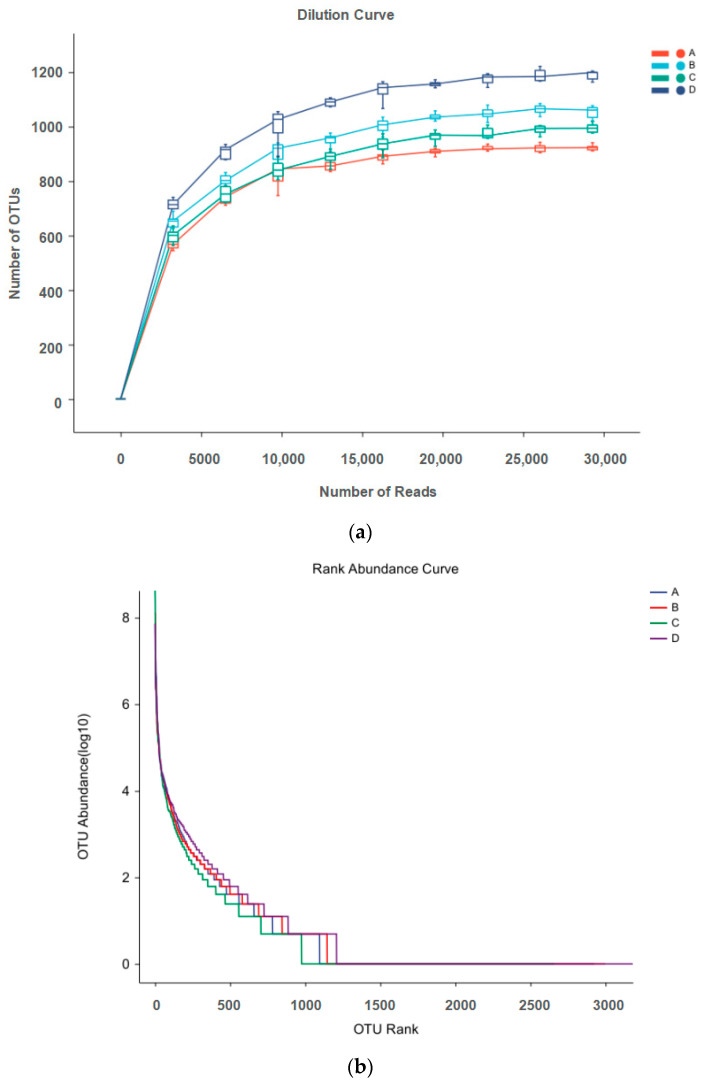
(**a**) Dilution curve of microbes in pigeon milk; (**b**) rank abundance curve of microbes in pigeon milk. Group A drank normal water; Group B was supplemented with 3 × 10^6^ CFU/mL *E. faecium*; Group C was supplemented with 2 × 10^7^ CFU/mL *B. subtilis*; Group D was supplemented with 3 × 10^6^ CFU/mL *E. faecium* and 2 × 10^7^ CFU/mL *B. subtilis*.

Microbial rarefaction curves were used to indicate alpha diversity reaching saturation in all samples. In this study, the number of OTUs increased exponentially before 5000 reads and achieved a close-to-horizontal state with increasing number of reads, suggesting that the sequencing data for each sample sufficiently reflected intact bacterial diversity, which covered 97% of the pigeon milk microbial strains (Figure 1a). The rank abundance curve (Figure 1b) shows that microbes of pigeon milk exhibited great bacterial richness and a high degree of evenness.

**Figure 2:**
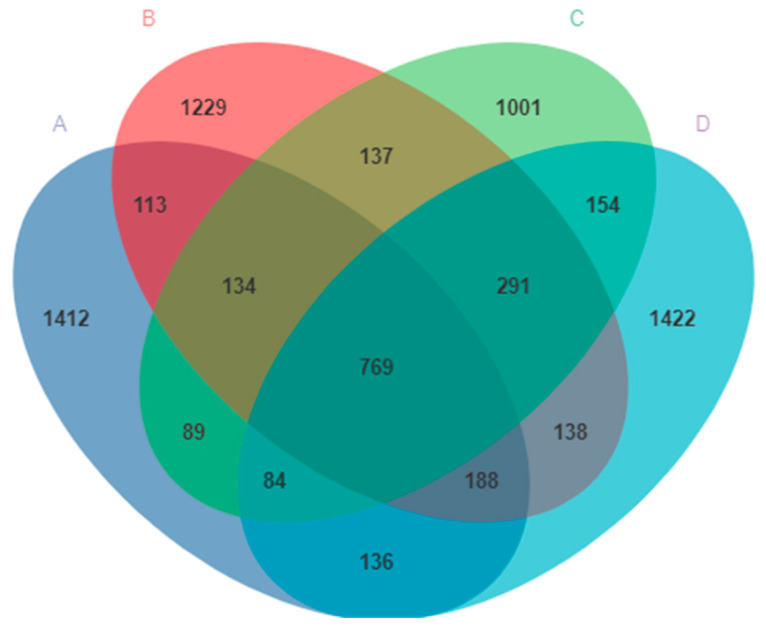
The ASVs clustering of microbes in pigeon milk. Group A drank normal water; Group B was supplemented with 3 × 10^6^ CFU/mL *E. faecium*; Group C was supplemented with 2 × 10^7^ CFU/mL *B. subtilis*; Group D was supplemented with 3 × 10^6^ CFU/mL *E. faecium* and 2 × 10^7^ CFU/mL *B. subtilis*.

The V3–V4 region of the 16S rDNA genes was sequenced from the pigeon milk collected from all four groups. A total of 1,670,381 sequences were obtained. After sequencing, errors were removed and chimeric filtering performed, so that 999,936 sequences remained. A total of 8140 OTUs were then obtained and annotated at the domain level, according to 99% sequence similarity. The exact numbers of OTUs obtained were 1412, 1229, 1001, and 1422 for Group A, Group B, Group C, and Group D, respectively (Figure 2). There were 769 OTUs common to all four groups, and the number of OTUs in group D was higher than that in any other groups (Figure 2).

**Figure 3:**
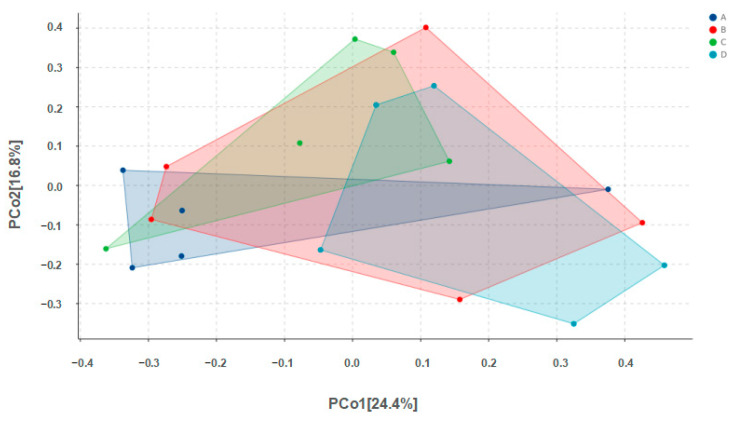
PCoA analysis of microbes in pigeon milk. Group A drank normal water; Group B was supplemented with 3 × 10^6^ CFU/mL *E. faecium*; Group C was supplemented with 2 × 10^7^ CFU/mL *B. subtilis*; Group D was supplemented with 3 × 10^6^ CFU/mL *E. faecium* and 2 × 10^7^ CFU/mL *B. subtilis*.

Alpha diversity results are shown in Table 4. No significant differences among groups were found for any of the indices. Values for Chao1 richness, observed species, Pielou’s evenness, the Shannon diversity index, and Simpson’s index for Group D were all higher than the corresponding values for Group A, but none of these differences were statistically significant (*p* > 0.05). Beta diversity results are shown in Figure 3.

**Figure 4:**
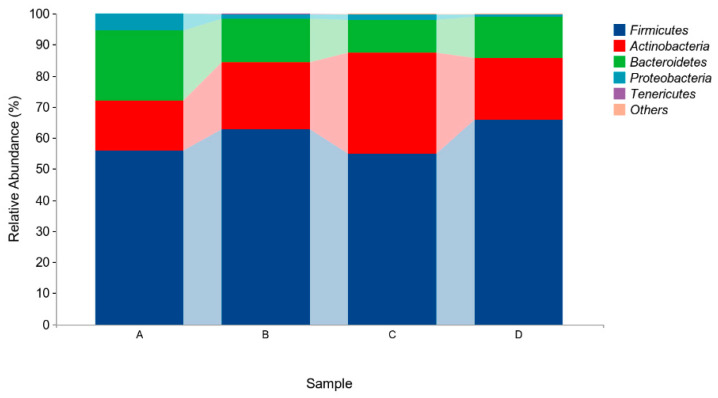
Relative abundances of species based on phylum classification level. Group A drank normal water; Group B was supplemented with 3 × 10^6^ CFU/mL *E. faecium*; Group C was supplemented with 2 × 10^7^ CFU/mL *B. subtilis*; Group D was supplemented with 3 × 10^6^ CFU/mL *E. faecium* and 2 × 10^7^ CFU/mL *B. subtilis*.

**Figure 5:**
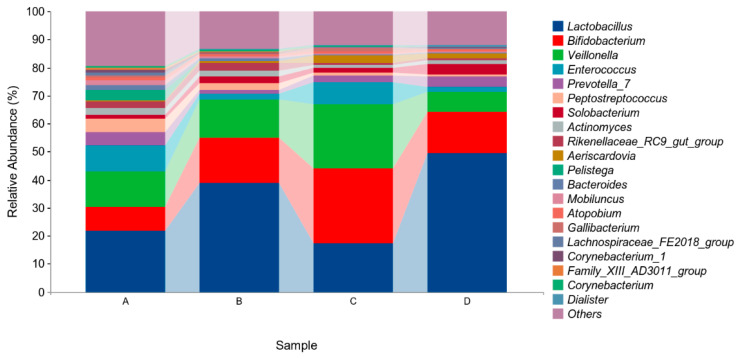
Relative abundances of species based on genus classification level. Group A drank normal water; Group B was supplemented with 3 × 10^6^ CFU/mL *E. faecium*; Group C was supplemented with 2 × 10^7^ CFU/mL *B. subtilis*; Group D was supplemented with 3 × 10^6^ CFU/mL *E. faecium* and 2 × 10^7^ CFU/mL *B. subtilis*.

The above results indicated that four different phyla were identified in the microflora of pigeon milk. At the phylum level, *Firmicutes*, *Actinobacteria*, and *Bacteroidetes* were the most abundant bacteria. The levels of *Firmicutes* in Group B and Group D were higher than those in in Group A and Group C, but not significantly (*p* > 0.05, Figure 4, Table 5). At the genus level, *Lactobacillus*, *Bifidobacterium*, *Veillonella* and *Enterococcus* were the four main genera identified (Figure 5, Table 6). The abundance of *Lactobacillus* was higher in Group B and Group D than in Group A and Group C, but not significantly (*p* > 0.05). The abundances of *Bifidobacterium* and *Veillonella* in Group C were higher in all groups but not significantly (*p* > 0.05).

## 4. Discussion

Pigeons are altricial birds. Pigeon squabs are unable to feed by themselves for a period of time after hatching, so they need to be fed with pigeon milk by parental birds [31]. Nutritional regulation in parental pigeons and the ingredients of crop milk are both important factors affecting the growth and development of squabs [26,32]. Probiotics are living microorganisms that beneficially affect host animals by modulating the gut microbiota, reducing disease risk, and improving growth performance [33,34]. *E. faecium*, which has been reported as a probiotic, normally colonizes in the gut. It has been found to exert a positive impact on poultry by improving growth performance, reducing ammonia emission, and preventing *Salmonella* infection [14,35]. *B. subtilis* is now established as a prominent probiotic species that is known to promote nutritional digestion and absorption [36]. In poultry breeding, it is always used as a growth promoter to increase digestibility and growth performance in broilers [36]. However, there have been few reports on the effects of using *E. faecium* or *B. subtilis* as supplements in the drinking water of parental pigeons in order to promote performance, immunity, and microorganisms in pigeon milk.

Many studies have confirmed that probiotics can improve growth performance by promoting enzymatic digestion through the provision of nutrients, vitamins, enzymes, and necessary growth factors to the host [8,37]. However, in this study, supplementation of the drinking water of parental pigeons with *E. faecium* and *B. subtilis* had no impact on the growth performance of their squabs. Similarly, Wen et al. (2022) also reported that a diet with *C. butyricum* had no impact on growth performance [26]. Other studies have found that supplementation with *C. butyricum* had no impact on growth ability in broilers and piglets [38,39]. Pigeon milk contains a large number of proteins, including active enzymes and immunoglobulins [31]. In one previous study, broilers fed a diet supplemented with *Bacillus* exhibited significantly higher enzyme activities (lipase, trypsin, and amylase) in their intestinal content, suggesting that supplementation with probiotics promoted digestion and absorption of nutrients; in addition, the serum concentration of IgA was significantly higher in the *Bacillus*-treated group [40]. The results of the present study indicated that supplementation of the water of parental pigeons with *E. faecium* and *B. subtilis* did not significantly promote the activities of lipase, trypsin, or amylase in the pigeon milk, while concentrations of IgA and IgG in the pigeon milk were significantly higher in the treated group. As previously reported, probiotics have a beneficial impact on the immune system by increasing production of different cytokines, activating immune cells, and increasing systemic immune response [41]. The IgA antibody plays a crucial role in immunity, especially with respect to humoral adaptive immune response. It can bind to pathogens and prevent them from invading by means of a noninflammatory process [41]. Probiotics can also activate IgA cycles and maintain the health of mucosal sites. In addition, probiotics may promote the production of IgG antibodies and the activation of IFNγ [42]. In the present study, we found that supplementation of the drinking water of parental pigeons with *E. faecium* and *B. subtilis* significantly increased the concentrations of IgA and IgG in pigeon milk, but the impact on the enzyme activities in pigeon milk and growth performance in squabs was found to be not significant.

Microbiota often exist in the intestines and milk of animals in a symbiotic relationship with their host [43,44,45]. Pigeon milk contains many microorganisms in a complex environment that could promote initial microorganism colonization and have an important impact on squab growth [2,46]. Microbiota diversity was found to be higher in the crop than in the small intestine and rectum [47]. *Firmicutes* and *Proteobacteria* were found to be the dominant phyla in the crops of chickens, while the dominant phyla in the crops of pigeons were reported as *Proteobacteria* and *Bacteroidetes* [47,48]. The microbial characteristics of pigeon milk acquired from squabs include eight phyla; amongst these, *Firmicutes*, *Actinobacteria*, and *Bacteroidetes* have the most microbiota [2]. In the present study, the dominant phyla in pigeon milk were found to be *Firmicutes*, *Actinobacteria*, and *Bacteroidetes*. In humans, *Firmicutes* was associated with energy and the absorption of nutrients [49]. *Actinobacteria* could produce substances which function as antibiotics [50]. Supplementation with *E. faecium* and *B. subtilis* might therefore improve digestion and immunity in squabs that consume pigeon milk from parental birds whose diet is so supplemented.

The main genera were *Lactobacillus*, *Bifidobacterium*, *Veillonella*, and *Enterococcus*. Compared with the control group, the group supplemented with *E. faecium* and *B. subtilis* had higher proportions of *Lactobacillus* and *Bifidobacterium*. Ding et al. (2020) reported that *Lactobacillus*, *Enterococcus*, *Veillonella*, and *Bifidobacterium* were the main microbiota in pigeon milk at the genus level [2]. *Lactobacillus* has been shown to stimulate both innate and acquired immune response in many species. Du et al. (2022) showed that *Lactobacillus* improved disease resistance in shrimp by regulating the nutritional immune response [51]. In humans, administration of *Lactobacillus* isolated from breast milk improved host immunity in adults through increased levels of immunoglobulins and numbers of immune cells [52,53]. In addition, *Lactobacillus* and *Bifidobacterium* have been shown to have beneficial affects on intestinal immunity by increasing the levels of IgA levels and other immunoglobulin-secreting cells [54]. *Lactobacillus* and *Bifidobacteria* have also been detected in the breast milk of human mothers in the first year of their child’s life, indicating roles in infant growth [55,56]. In mice, *Lactobacillus* has been found to protect epithelial cells not exposed to viruses [57]. In the present study, the groups of pigeons with higher proportions of *Lactobacillus* and *Bifidobacterium* also presented higher immunoglobulin levels in their pigeon milk. In brief, immunity may be promoted through supplementation with *E. faecium* and *B. subtilis*.

## 5. Conclusions

Our results showed that supplementation of the drinking water of parental pigeons with *E. faecium* and *B. subtilis* had a positive impact on the immunoglobulin levels in pigeon milk. Under experimental conditions, we found that supplementation with 3 × 10^6^ CFU/mL *E. faecium* and 2 × 10^7^ CFU/mL *B. subtilis* could improve concentrations of IgA and IgG, suggesting that these probiotics improve the ability of squabs to resist disease. In terms of microbiological compositions, at the phylum level, *Fimicutes*, *Actinobacteria*, and *Bacteroidetes* were the three main phyla identified. At the genus level, *Lactobacillus*, *Bifidobacterium*, *Veillonella*, and *Enterococcus* were the four main genera identified. These results suggest that *E. faecium* and *B. subtilis* could be used as probiotics in the pigeon industry.

## Figures and Tables

**Table 1:** Effects of *Enterococcus faecium* and *Bacillus subtilis* on the growth performance of squabs.

Items	Groups ^1^				Pooled SEM	*p*-Value
	Group A	Group B	Group C	Group D		
Body weight, g						
at Day 1	16.32	15.09	15.58	15.52	0.20	>0.05
at Day 2	51.89	43.94	48.85	51.87	1.39	>0.05
at Day 12	266.11	282.49	291.92	291.92	4.57	>0.05
ADG ^2^, g/d						
Days 1–12	20.82	22.28	23.03	23.03	0.38	>0.05

^1^ Group A drank normal water; Group B was supplemented with 3 × 10^6^ CFU/mL *E. faecium*; Group C was supplemented with 2 × 10^7^ CFU/mL *B. subtilis*; Group D was supplemented with 3 × 10^6^ CFU/mL *E. faecium* and 2 × 10^7^ CFU/mL *B. subtilis*. ^2^ ADG represents average daily gain.

**Table 2:** Effects of *Enterococcus faecium* and *Bacillus subtilis* on enzymatic activity in pigeon milk.

Items	Groups ^1^				Pooled SEM
	Group A	Group B	Group C	Group D	
Lipase (U/g)	144.89 ^ab^	111.41 ^b^	138.77 ^a^	185.12 ^a^	7.59
Trypsin (ng/mg)	78.32 ^ab^	63.32 ^b^	80.80 ^ab^	106.01 ^a^	4.92
Amylase (U/g)	122.82 ^ab^	98.23 ^b^	125.04 ^ab^	172.42 ^a^	8.49

^1^ Group A drank normal water; Group B was supplemented with 3 × 10^6^ CFU/mL *E. faecium*; Group C was supplemented with 2 × 10^7^ CFU/mL *B. subtilis*; Group D was supplemented with 3 × 10^6^ CFU/mL *E. faecium* and 2 × 10^7^ CFU/mL *B. subtilis*. ^a,b^ Different small-letter superscripts in the same row indicate *p* < 0.05 for the means.

**Table 3:** Effects of *Enterococcus faecium* and *Bacillus subtilis* on immunoglobulin levels in pigeon milk.

Items	Groups ^1^				Pooled SEM
	Group A	Group B	Group C	Group D	
IgA (μg/mL)	15.68 ^b^	23.21 ^b^	26.15 ^ab^	35.86 ^a^	1.87
IgG (μg/mL)	1124.56 ^b^	1563.30 ^b^	1752.63 ^ab^	2381.52 ^a^	118.67

^1^ Group A drank normal water; Group B was supplemented with 3 × 10^6^ CFU/mL *E. faecium*; Group C was supplemented with 2 × 10^7^ CFU/mL *B. subtilis*; Group D was supplemented with 3 × 10^6^ CFU/mL *E. faecium* and 2 × 10^7^ CFU/mL *B. subtilis*. ^a,b^ Different small-letter superscripts in the same row indicate *p* < 0.05 for the means.

**Table 4:** Effects of *Enterococcus faecium* and *Bacillus subtilis* supplementation on alpha diversity indices for microbes in pigeon milk.

Groups ^1^	Chao1 Richness	Faith’s PD	Good’s Coverage	Observed Species	Pielou’s Evenness	Shannon Diversity Index	Simpson’s Index
A	923.84	43.24	0.99	806.00	0.62	6.01	0.93
B	1057.60	41.08	0.99	829.92	0.61	5.99	0.91
C	993.67	40.29	0.99	832.70	0.57	5.49	0.88
D	1193.33	40.11	0.99	1007.82	0.68	6.77	0.96
*p*-value	0.37	0.85	0.45	0.49	0.18	0.42	0.40

^1^ Group A drank normal water; Group B was supplemented with 3 × 10^6^ CFU/mL *E. faecium*; Group C was supplemented with 2 × 10^7^ CFU/mL *B. subtilis*; Group D was supplemented with 3 × 10^6^ CFU/mL *E. faecium* and 2 × 10^7^ CFU/mL *B. subtilis*.

**Table 5:** Relative abundances of species based on phylum classification level (abundance of the phylum is expressed as a percentage).

Items	Groups ^1^				Pooled SEM	*p*-Value
	Group A	Group B	Group C	Group D		
*Firmicutes*	55.88	62.72	54.75	65.90	0.05	>0.05
*Actinobacteria*	16.24	21.70	32.50	19.59	0.04	>0.05
*Bacteroidetes*	22.50	13.70	10.82	13.37	0.03	>0.05
*Proteobacteria*	5.20	1.70	1.73	0.96	0.01	>0.05

^1^ Group A drank normal water; Group B was supplemented with 3 × 10^6^ CFU/mL *E. faecium*; Group C was supplemented with 2 × 10^7^ CFU/mL *B. subtilis*; Group D was supplemented with 3 × 10^6^ CFU/mL *E. faecium* and 2 × 10^7^ CFU/mL *B. subtilis*.

**Table 6:** Relative abundances of species based on genus classification level (abundance of the genus is expressed as a percentage).

Items	Groups ^1^				Pooled SEM	*p*-Value
	Group A	Group B	Group C	Group D		
*Lactobacillus*	21.73	38.88	17.37	49.40	0.06	>0.05
*Bifidobacterium*	8.63	15.95	26.55	14.82	0.04	>0.05
*Veillonella*	12.47	13.76	23.06	6.99	0.03	>0.05
*Enterococcus*	9.25	1.88	7.72	1.89	0.02	>0.05

^1^ Group A drank normal water; Group B was supplemented with 3 × 10^6^ CFU/mL *E. faecium*; Group C was supplemented with 2 × 10^7^ CFU/mL *B. subtilis*; Group D was supplemented with 3 × 10^6^ CFU/mL *E. faecium* and 2 × 10^7^ CFU/mL *B. subtilis*.

## Data Availability

The sequencing data were submitted to the NCBI SRA database (accession number: PRJNA 1042862). They are accessible with the following link https://www.ncbi.nlm.nih.gov/sra/PRJNA1042862, assessed on 22 November 2023.
